# The Effect of Self-Efficacy in Self-Management on Diabetes Distress in Young People with Type 2 Diabetes

**DOI:** 10.3390/healthcare9121736

**Published:** 2021-12-15

**Authors:** Pao-Yu Lin, Tzu-Ying Lee, Chieh-Yu Liu, Yann-Jinn Lee

**Affiliations:** 1 School of Nursing, National Taipei University of Nursing and Health Sciences, Taipei 112303, Taiwan; s162@mail.mkc.edu.tw; 2 Department of Nursing, MacKay Junior College of Medicine, Nursing and Management, Taipei 112021, Taiwan; 3 Department of Health Care Management, National Taipei University of Nursing and Health Sciences, Taipei 112303, Taiwan; chiehyu@ntunhs.edu.tw; 4 Laboratory of Molecular Medicine, Department of Pediatric Endocrinology, MacKay Children’s Hospital, Taipei 104217, Taiwan; yannlee@mmh.org.tw; 5 Department of Medical Research, MacKay Memorial Hospital, Taipei 104217, Taiwan

**Keywords:** diabetes management, distress, self-efficacy, type 2 diabetes, youth

## Abstract

To understand the relationship among glycemic control, self-efficacy in diabetes management, and diabetes distress in young people with type 2 diabetes, a cross-sectional descriptive study with convenience sampling was designed. A total of 60 young people who had type 2 diabetes (T2D), with 24 (40%) males and 36 (60%) females were included. The mean age was 17.2 and ranged from 10.5 to 24.5 years, and they completed a Perceived Diabetes Self-Management Scale, the Problem Areas in Diabetes Scale and their pharmacologic management and life adjustment. Glycated hemoglobin (HbA1c) was routinely drawn before the outpatient visit. HbA1c and diabetic distress were positively correlated. Self-efficacy was negatively correlated with HbA1c and diabetic distress. In the hierarchical multiple regression analysis, only the duration of illness and self-efficacy remained significant in the final model. The variance for the overall model was 64%, with self-efficacy alone explaining 30% of the variance. In addition, 31.6% of participants had extremely high levels of psychological distress. Conclusions: T2D is an early onset chronic disease, and the young people may have had other health problems, which made the diabetes management a complex process. Nursing staff should regularly assess both the confidence and ability to manage treatment regimen of young people with type 2 diabetes and their psychological distress.

## 1. Introduction

The global incidence and prevalence of youth-onset type 2 diabetes (T2D) has increased dramatically over the past 20 years, in parallel with the increase in the rate of obesity [1,2]. Based on a nationwide longitudinal study in Taiwan, the incidence rate of youth-onset T2D was 63 cases per 100,000 young people and was more prevalent than type 1 diabetes in a population of young age [3,4]. Unlike the adult-onset of T2D, youth-onset of T2D has a more rapid progressive decline in beta cell function and has an increased risk of complications related to long-term hyperglycemia and other atherogenic risk factors at an earlier age [1,5]. Some of the risk factors for youth-onset T2D include being overweight or obese, a family history of diabetes and being female. The risk factors and characteristics of young people with T2D add to the complexity of self-management [6]. Thus, care for youth-onset of T2D deserves more attention from healthcare providers.

Diabetes distress refers to negative emotions (e.g., frustration, guilt, hopelessness, anger and fear), that arise from living with and managing diabetes [7]. Diabetes management for young people with T2D involves lifestyle adjustments, including regular exercise, weight loss, dietary control, blood glucose monitoring to improve or achieve glycemic control and possible pharmacologic treatment [1,8]. However, lack of support for physical activity and nutritional self-efficacy and higher BMI are some of the risk factors for increased depressive symptoms in young people with T2D [2]. The transition from adolescence to early adulthood involves dramatic physical, psychological and social developmental changes [9]. Developing self-identity and seeking independence are these young people’s important growth tasks [10,11]. When making healthy lifestyle adjustments and self-management behaviors required by T2D, the unique psychosocial development and cultural environment among this young age group may result in difficulties. Previous qualitative studies also found that young people with T2D diabetes had difficulties in identifying and accepting themselves as individuals with T2D diabetes. They felt different from their peers, choosing to hide the health condition from their peers [12,13,14]. Furthermore, duration of T2D has a positive relationship with diabetes distress [15]. Because higher depressive symptoms over time, associated with chronic diabetes distress, were found in young people with diabetes [16], surveillance of mental health is necessary [15,17].

Self-efficacy is a measure of an individual’s level of belief or confidence in his/her ability to complete tasks and achieve goals [18,19]. It is thought that people’s self-efficacy beliefs can influence the health-related choices they make, the health-related goals they set for themselves, and the amount of effort they expend to reach their goals [20]. Many studies have shown that young people with T1D who have high self-efficacy confidence in their abilities can persevere more when facing self-care challenges [21,22]. High HbA1c and low self-efficacy have been shown to predict high diabetes distress [23,24].

To date these studies that refer to self-management and emotional care have primarily focused on young people with T1D diabetes, including in Taiwan [25], which currently faces an increasing number of young people with youth-onset T2D. Because the young people with T2D need to undertake the complex task of adjusting and changing their original lifestyles, more research is needed to understand diabetes-related distress and its relationship with self-efficacy in diabetes management among this group.

Thus, this study aimed to examine the influences of glycemic control, diabetes management, and self-efficacy on diabetes distress among young people with T2D after adjusting for the potential risk factors, including participants’ characteristics such as being overweight or obese, a family history of diabetes and sex. The research hypotheses of this study are as follows: 1. The lower the self-efficacy of diabetes in young people, the higher the diabetic distress is and 2. The lower the diabetic distress in young people, the better glycemic control is.

## 2. Materials and Methods

### 2.1. Design, Setting and Participants

A cross-sectional study with convenience sampling was designed. All participants were recruited from the pediatric diabetes outpatient clinic of a medical center in northern Taiwan from February to November 2019.

The World Health Organization uses the term “young people” to cover “adolescents” (10–19 years age group) and “youth” (15–24 years age group) [9]. Therefore, the inclusion criteria were young people who (1) were 10–24 years of age, (2) were currently enrolled in school, (3) had a confirmed diagnosis of type 2 diabetes and (4) had Mandarin or Taiwanese speaking and reading abilities. Exclusion criteria were: (1) Precocious puberty, (2) the presence of other major illnesses and mental dysfunction. The sample size determination of this study was based on multiple linear regression; assuming the medium effect size 0.2, α = 0.05, power = 0.8, by using G*power version 3.1 [26], the estimated sample size was 62. Although there were 2 participants who refused to participate in this study, the effect size was 1.74 based on the R^2^ of our final step (R^2^ = 0.635). The power of this study was greater than 80.

### 2.2. Diabetes Distress

Psychological distress in young people with diabetes was measured by the Problem Areas in Diabetes Scale (PAID) [27,28]. The scale consisted of 20 items, scored on a 5-point Likert scale, with 0 indicating “not a problem” and 4 indicating a “serious problem”. These scores were added and multiplied by 1.25 to get a total score between 0 and 100. Patients with scores of 40 or higher were considered to have “significant diabetes distress” that required special attention [17,29]. The scale had a Cronbach’s α of 0.95. Huang, Courtney, Edwards and McDowell translated the scale into the Chinese Version of the Problem Area in Diabetes (PAID-C), with a retest reliability of 0.83 [30]. The scale was used after obtaining consent, and Cronbach’s α was 0.92 in this study.

### 2.3. Glycemic Control

Glycemic control was indicated by HbA1c. Based on the guidelines from the American Diabetes Association [1], a treatment target of HbA1c less than 7% is recommended. In this study, HbA1c less than 7% was considered an indicator of good glycemic control. A blood draw for HbA1c was routinely done in the diabetes outpatient clinic 3–5 days prior to the subsequent visit. These data were collected as part of their medical records.

### 2.4. Self-Efficacy in Diabetes Management

Self-efficacy in diabetes management was measured using the Perceived Diabetes Self-Management Scale (PDSMS) developed by Wallston [20]. The scale consisted of 8 items on a Likert 5-point scale, corresponding to “1: strongly disagree” and “5: strongly agree”, respectively. Items 1, 2, 6 and 7 were scored in reverse, for a total score from 8 to 40. Higher scores indicated that the patient had more confidence in diabetes self-management. Cronbach’s α was 0.83 in the previous study [20]. The Chinese-Perceived Diabetes Self-Management Scale (C-PDSMS) was translated by Lin and colleagues [31]. Cronbach’s α was 0.93 and the test-retest reliability was 0.97 in Lin et al.’s study. The scale was used after receiving consent, and Cronbach’s α was 0.89 in this study.

### 2.5. Demographic and Diabetes Management Information

Demographic data included the patient’s age, gender and education, age at T2D onset, duration of T2D, and the educational level of their primary caregiver. Additionally, height and weight, type of treatment, and other health problems (i.e., hypertension, hyperlipidemia, fatty liver, metabolic syndrome) were obtained from the medical records. Body Mass Index (BMI) was computed using the formula: weight in kilograms/height in meters^2^. Taiwan’s Ministry of Health and Welfare recommended for ages 18 and older a BMI between 18.5 and <24 as the normal range, a BMI of ≥24 and ≤27 as overweight, and a BMI of ≥27 as obese. For ages less than 18, a BMI-for-age percentiles growth chart based on the anthropometrics of the age population in Taiwan was used to decide their BMI status [32].

Diabetes management information included types of medications taken (oral hypoglycemic agents, insulin injections and oral hypoglycemic agents combined with injections), frequency of medications missed per week (including oral hypoglycemic agents and insulin injections), frequency of exercise per week and exercising alone versus with others. In addition, 13 recommended dietary adjustments that were routinely taught to the patients by the diabetes educator in the diabetes outpatient clinic were listed for the participants to mark; thus, the number of dietary adjustment recommendations they followed also were collected.

### 2.6. Procedures

Recruitment of participants began after all study procedures were approved by the Institutional Review Board of the hospital. Each participant and their parents were informed of the HbA1c values and were routinely tested 3–5 days pre-visit and by the physician on the day of the clinic visit. After completing the physician visit, the patient and the accompanying parent were approached and invited to participate by the investigator. Once they agreed to participate, a signed consent form was obtained from the participant older than 20 years old, or a signed assent from the participant younger than 20, in addition to the parent’s consent form. Subsequently, participants completed all the instruments, which were returned to the investigator.

### 2.7. Data Analysis

Data were processed using SPSS software version 25 (IBM Corp., Armonk, NY, USA) and were summarized as the mean and standard deviation for continuous variables and as proportions for categorical variables. A *t*-test, one-way analyses of variance and Pearson product-moment correlation were used, as appropriate, to analyze the association of participants’ characteristic diabetes-management variables and self-efficacy with diabetes distress. Significant participants’ characteristics and self-management variables were further added into the hierarchical multiple regression sequentially for adjustment. Thus, in step 1, the participants’ characteristics (age at T2D onset and duration of disease) were added to the model first, followed by HbA1c and diabetes-management terms (frequency of exercise and frequency of medications missed per week) in step 2, and self-efficacy in step 3 to identify the effects of these covariates on diabetes distress in T2D youths. No collinearity in the model was indicated by the variance inflation factor values of the covariates less than 10 [33].

## 3. Results

### 3.1. Descriptive Statistics

A total of 60 participants were included in the study. Demographic information is presented in Table 1. Their age ranged from 10.5 to 24.5 years. More than 80% of participants were either overweight or obese, and 85% had a family history of T2D. Thirty-one (51.7%) participants had 1 to 3 health problems besides T2D. The health problems included hyperlipidemia (52.4%), metabolic syndrome (26.2%), hypertension (14.3%) and fatty liver (7.1%). The mean HbA1c was 8.7%, and 60% of participants had HbA1c greater than 7%.

In terms of treatment, 5 participants (8.3%) needed no medication but were advised by the physician to adjust their lifestyle (including increased physical activity, weight loss and dietary control). More than half of the participants (53.3%) were additionally treated with oral hypoglycemic agents to lower blood sugar, 6.7% (*n* = 4) had to have insulin injections, one of them was normal weight and the other was obese, and their HbA1c was 12.2%, 13.7%, 14.8% and 8.7%. In addition, 31.7% had oral drugs combined with insulin injections. Among the 55 participants who needed to have medication treatments, only 8 participants reported good medication compliance. That is, about 85.5% of the participants who needed to have medication treatments missed at least once per week.

According to 13 dietary adjustment recommendations provided by the diabetes educator, the mean recommendations that 60 participants chose to follow was 3.8, ranging from 1 to 10. The common dietary adjustments were to “reduce the consumption of sugary drinks” (19.2%), followed by “eating less rice” (13.1%), “avoid purified starch” (12.2%), and “eating less fried food” (8.7%). More than half of the participants (68.3%) exercised less than 3 times a week, and the most common period of exercise was physical education class at school (35.2%), followed by weekend and holiday exercise (21.9%). Most exercises were done together with friends (45.5%), followed by solo exercise (33.8%). The primary caregivers were predominantly mothers (71.7%). Most primary caregivers had a high school education level or lower, and about 43% of the families were of middle-low socioeconomic status. The mean PDSMS was 28.8, ranging from 15–37, and the mean PAID was 33.9, ranging from 7.5 to 77.5, with 19 (31.6%) participants having a PAID above 40.

The association between characteristics of participants, study variables and diabetic distress was analyzed by t-tests and one-way ANOVA. Those who exercised 3 times or more per week had significantly higher self-efficacy (*t* = −3.22, *p* < 0.05) and lower distress (*t* = 2.43, *p* = 0.02) than those who exercised less than 3 times per week. There were no statistically significant differences in diabetes distress among individuals with T2D in terms of sex, stage of puberty, education level, BMI classification, family history of T2D and type of treatment (Table 1). The findings of Pearson product-moment correlation showed that duration of T2D, frequency of medications missed per week and HbA1c (*r* = 0.28, *p* = 0.03; *r* = 0.42, *p* = 0.001; *r* = 0.34, *p* = 0.009) were significant positive correlations with diabetes distress. Age at T2D onset and self-efficacy showed significant negative correlations with diabetes distress (*r* = −0.31, *p* = 0.17; *r* = −0.74, *p* < 0.001). The results of the bivariate correlations of study variables are presented in Table 2.

### 3.2. Relationship of Covariates and Diabetes Distress in Youth with T2D

Hierarchical multiple regression was used to analyze the variables that affected significant differences in diabetes distress. In step 1, age at T2D onset and duration of T2D were added into the model. Only duration of T2D was significantly related to diabetes distress (*β* = 0.29, *p* = 0.02). In step 2, HbA1c and self-management terms including frequency of medications missed per week and frequency of exercise were added into the model. The participants having longer duration of T2D and missing more doses of medications per week had higher diabetes distress (*β* = 0.26, *p* = 0.03; *β* = 0.30, *p* = 0.02). Last, after self-efficacy was added into the model, only duration of T2D (*β* = 0.22, *p* = 0.01) and self-efficacy (*β* = −0.63, *p* < 0.001) were significantly related to diabetes distress. Those with a longer duration of T2D and lower self-efficacy had the higher diabetes distress, regardless of age at T2D onset, HbA1c, frequency of medications missed per week and frequency of exercise per week. The third regression model explained a total of 64% of the variance in diabetes distress, with self-efficacy in diabetes management accounting for 29% of the variance alone (Table 3).

## 4. Discussion

The present study examined diabetes distress and its association with participants’ characteristics, diabetes management, glycemic control and self-efficacy variables in young people with T2D. Previous research found that without immediate intervention, diabetes distress in some young people with diabetes did not go away but instead transformed into chronic diabetes-related distress (DRD). Chronic DRD patients have been observed to have more psychological problems [16]. In the present study, there were 31.6% young participants with T2D having a PAID score above 40; these participants were considered as having significant diabetes distress that might require special attention [29]. Fisher et al. (2019) [17] suggested that discussion about the emotional side of diabetes intervention should be integrated into diabetes education and all clinical encounters newly diagnosed. Small group programs for individuals with high levels of diabetes distress can help the individual to share experiences and enhance emotion management skills.

With regard to individuals’ characteristics, average age at T2D onset was 13.6 years in this study. Type 2 diabetes typically occurs in adolescents at mid-puberty (for example, the mean age of diagnosis was 14 years) [34], most likely precipitated by the physiologic, but transient, pubertal insulin resistance aggravating the preexisting metabolic challenges of obesity. Puberty is a time of rapid and complex changes involving overlapping components: hormonal, physical and cognitive. Tanner Staging, also known as Sexual Maturity Rating (SMR), is an objective classification system that providers use to document and track the development and sequence of secondary sex characteristics of children during puberty [35]. It was developed by Marshall and Tanner while conducting a longitudinal study from the 1940s to the 1960s in England. In females, the normal onset of puberty ranges from 8 to 13 years old; menarche, the onset of menses, arrives on average at age 12.5 years, regardless of ethnicity, following thelarche on average by 2.5 years (range 0.5 to 3 years). Between Tanner Stage 2 and 3 breast development, females experience peak height velocity. African-American females have closer to 3 years between their thelarche and menarche. In males, the onset of puberty ranges from 9 to 14 years of age. The first secondary sexual characteristic visible is gonadarche [36,37]. According to the Tanner score, in our study, we classified under 16 years of age as prepubertal and those over 16 years of age as postpubertal. However, there was no statistically significant difference in young people with T2D between the two groups.

Most of our subjects had a family history of T2D, with oral hypoglycemic agents being the primary treatment for individuals. More than 50% of our participants had 1 to 3 health problems such as hyperlipidemia, metabolic syndrome and hypertension besides T2D. This finding further supports that individuals with youth-onset T2D tend to develop diabetes-related complications at a younger age, such as cardiovascular morbidity, which will make diabetes management more difficult [4,12]. In addition, in the present study there were no significant differences in diabetes distress between the sexes of our T2D young people. Our sex effect is different from a previous study that found higher levels of diabetes distress in adolescent girls with T1D than in adolescent boys [38], but it is consistent with the finding of another study on adults with T2D [15]. Furthermore, Islam et al. found that obese T2D patients had higher diabetes distress than the less obese ones [39]. However, in our study, more than 80% of the young patients were overweight or obese, and there was no significant BMI difference between male and female groups in the post hoc analysis (*Z* = 0.73, *p* = 465). It is inferred that the high homogeneity of the BMI in both boys and girls and in our entire group may also explain why sex and BMI did not show significant associations with psychological distress.

As for the findings of diabetes management and glycemic control, Mulvaney et al. indicate that most patients are diagnosed during adolescence, which is an important stage in the development of peer friendships and the consolidation of self-identity [6]. Therefore, peers can play an important role in the successful self-management of youth-onset T2D. For example, youths with T2D tend to maintain sustained physical activity when they are with peers [40]. Although in the present study most of the young people with T2D reported that they exercised with their classmates, the most common time to exercise was in school and only during physical education classes; more than half of the young people exercised less than 3 times a week. This also indicated that many participants still had not developed independent and regular exercise habits and had higher distress in diabetes management. Furthermore, despite the fact that the healthcare providers in the diabetes outpatient clinics provide routine health education on physical activities, diet and medication regimens, in the present study 85.5% of the young patients who needed medication treatments missed medication once or more per week, and the more they missed, the higher the distress they experienced. In addition, we found that the 13 dietary adjustment recommendations provided by the healthcare teams were not completely followed by the participants. This also was consistent with our other descriptive findings that 60% of participants had poor glycemic control (HbA1c ≥ 7%). It is thought that young people tend to engage in risky or less responsible behaviors as part of their identity explorations [10,11] and that they have difficulties in implementing lifestyle modifications for diabetes management [41]. The actual reasons for poor lifestyle adjustment and low medication adherence should be further understood.

In the final model of hierarchical regression analysis, duration of T2D had a positive relationship with diabetes distress, which is consistent with previous research carried out with T2D adults [15]. It is possible that for the young people with a long duration of T2D, they continuously needed to go through different challenges from early adolescence to late adolescence and even to emerging adulthood. The challenges derived from the environment (i.e., family, school and peers) might make the social situation complicated and the lifestyle adjustment more difficult. For example, Nouwen et al. found that, compared to younger adolescents, older adolescents attached more importance to peer acceptance and activities; thus, they might feel distressed about their disease and the self-management regimen [24].

In the present study, self-efficacy predicted diabetes distress after adjusting for all the other variables in the third model, with a 30% variance explained alone. Previous studies found that people’s positive self-efficacy beliefs achieved good diabetes control through influencing their health-related choices and the efforts they made to reach their goals, and further predicted diabetes distress in T1D youth [20,21,22,23,24]. Our finding supports that high self-efficacy is also an important protector for diabetes distress in T2D young patients, but our descriptive data, in fact, showed that many participants had poor adherence to treatment regimens. Mulvaney et al. suggested that social aspects, including peers, family members and school environment, may influence the attitudes and self-management of young patients with T2D [6]. Young people feel frustrated with life changes and lack of normalcy associated with diabetes. In another qualitative study, both the T2D adolescent and the mother found it difficult to change long-standing eating habits, as well as other treatment regimens [12]. Healthcare providers may need to work more closely with the individual with T2D diabetes and the family to have a long-term plan with realistic goals for them to achieve.

This study had three limitations. First, the study had a small sample size. Due to the characteristics of T2D, young patients are not regularly seen or diagnosed in the outpatient clinic, which made recruitment difficult. Second, because the data collection was limited to one setting in Taiwan, these results might not be generalizable to other young people with T2D. Third, the study did not collect the sex hormones. It could not accurately analyze the relationship between sex hormones and emotional distress. Finally, the PDSMS we used was based on the participants’ beliefs about and confidence in diabetes management, but we were not able to measure their abilities from their daily practice. Future studies could include more young patients with T2D and focus on understanding their knowledge of how to manage T2D and their ability to do so, as well as their resources, and barriers encountered.

## 5. Conclusions

Based on the findings of our study, many participants had a family history of T2D, were overweight or obese, had other health problems, and had poor diabetes self-management in exercise and medication adherence. If they were not confident in their ability to manage the diabetes, they would have high emotional distress and the whole situation could be worsened. Because youth-onset T2D becomes a childhood chronic disease, once diagnosed, healthcare providers will see the young patients for years, from childhood through their emerging adulthood—a unique and fast transitional period of physical, cognitive and psycho–social development. Thus, it is important to work with these young people and their families in the early stages to develop an age-appropriate comprehensive care plan to increase their self-efficacy, which not only includes strategies of physical activity enhancement, dietary control, obesity control and medication adherence, but also emotional support related to diabetes distress, to improve their health. Regularly assessing both the confidence and practice of young people with T2D to manage a treatment regimen and their psychological distress is greatly needed.

## Figures and Tables

**Table 1:** Characteristics of participants, study variables and their association with diabetic distress (N = 60).

Variables	*n* (%)/*M* ± *SD*	Diabetes Distress		
		*M* ± *SD*	*t/F*	*p*
Age	17.2 ± 3.2			
Stage of puberty			1.104	0.27
Prepuberty (<16 years)	22 (36.7)	37.05 ± 19.02		
Postpuberty (≥16 years)	38 (63.3)	32.07 ± 15.42		
Age at T2D onset	13.6 ± 2.6			
Duration of T2D (years)	3.5 ± 2.7			
Sex			0.22	0.83
Male	24 (40.0)	34.5 ± 16.8		
Female	36 (60.0)	33.5 ± 17.1		
Education Level			−0.31	0.76
≤High school	42 (70.0)	33.5 ± 17.3		
≥College	18 (30.0)	34.9 ± 16.3		
Classification of BMI			0.35	0.70
Normal	10 (16.7)	37.6 ± 14.2		
Overweight	8 (13.3)	35.2 ± 17.7		
Obese	42 (70)	32.8 ± 17.5		
Having family history of T2D	51 (85.0)	32.7 ± 16.8	1.35	0.18
Having Other diagnosis	31 (51.7)			
HbA1c (%)	8.7 ± 2.7		−2.00	0.05
≤7.0	24 (40.0)	28.7 ± 14.5		
>7.0	36 (60.0)	37.4 ± 17.6		
Types of treatments			0.90	0.45
Lifestyle adjustment only	5 (8.3)	25.5 ± 8.2		
Oral drugs	32 (53.3)	33.9 ± 16.5		
Insulin injection	4 (6.7)	44.1 ± 26.7		
Oral drugs combined with insulin injection	19 (31.7)	33.9 ± 16.9		
Frequency of medications missed per week (times)	2.8 ± 2.8			
Number of dietary adjustments followed	3.8 ± 2.1			
Frequency of exercise per week (times)			2.43	0.02
<3	41 (68.3)	37.3 ± 17.1		
≥3	19 (31.7)	26.4 ± 13.9		
Primary caregiver			1.02	0.31
Father	17 (28.3)	37.4 ± 16.3		
Mother	43 (71.7)	32.5 ± 17.1		
Education Level of primary caregiver			1.13	0.26
≤High school	38 (63.3)	35.8 ± 16.1		
≥College	22 (36.7)	30.7 ± 18.0		
Self-efficacy (PDSMS)	28.8 ± 6.1			
Diabetes distress (PAID)	33.9 ± 16.8			

Notes. PDSMS = Perceived Diabetes Self-Management Scale, PAID = Problem Areas in Diabetes Scale.

**Table 2:** Bivariate correlations of study variables (N = 60).

	2	3	4	5	6	7
Age at T2D onset	−0.12	−0.04	0.24	−0.09	0.16	−0.16
2. Duration of T2D		0.05	0.05	−0.01	−0.13	0.31 *
3. Frequency of medications missed per week			−0.13	0.37 **	−0.38 **	0.42 **
4. BMI				−0.16	0.09	−0.09
5. HbA1c					−0.26 *	0.34 **
6. Self-efficacy						−0.74 ***
7. Diabetes distress						1

Notes. * *p* < 0.05, ** *p* < 0.01, *** *p* < 0.001.

**Table 3:** Hierarchical multiple regression analysis of predictors for diabetes distress (N = 60).

Predictors	*B*	*SE*	*β*	*t*	*p*	*R* ^2^	Δ*R*^2^	VIF
Step 1						0.11	0.11	
Age at T2D onset (year)	−0.79	0.81	−0.12	−0.97	0.33			1.01
Duration of T2D (years)	1.82	0.78	0.29	2.33	0.02			1.01
Step 2						0.34	0.23	
Age at T2D onset (year)	−0.11	0.77	−0.02	−0.14	0.89			1.19
Duration of T2D (years)	1.60	0.70	0.26	2.29	0.03			1.04
HbA1c (%)	1.43	0.76	0.23	1.89	0.06			1.17
Frequency of medications missed per week (times)	1.79	0.72	0.30	2.47	0.02			1.19
Frequency of exercise per week (times)	−7.26	4.39	−0.20	−1.66	0.10			1.23
<3								
≥3								
Step 3						0.64	0.29	
Age at T2D onset (year)	−0.03	0.58	−0.01	−0.06	0.96			1.19
Duration of T2D (years)	1.36	0.53	0.22	2.60	0.01			1.04
HbA1c (%)	0.80	0.58	0.13	1.39	0.17			1.20
Frequency of medications missed per week (times)	0.76	0.57	0.13	1.33	0.19			1.29
Frequency of exercise per week (times)	−0.93	3.44	−0.03	−0.27	0.79			1.33
<3								
≥3								
Self-efficacy	−1.74	0.27	−0.63	−6.52	<0.001			1.34

## Data Availability

The data are not publicly available due to privacy or ethical reasons.

## References

[1] American Diabetes Association (2020). 13. Children and Adolescents: Standards of Medical Care in Diabetes—2021. Diabetes Care.

[2] Kao K.T., Sabin M.A. (2016). Type 2 diabetes mellitus in children and adolescents. Aust. Fam. Physician.

[3] Chen M.H., Lan W.H., Hsu J.W., Huang K.L., Su T.P., Li C.T., Lin W.C., Tsai C.F., Tsai S.J., Lee Y.C. (2016). Risk of Developing Type 2 Diabetes in Adolescents and Young Adults with Autism Spectrum Disorder: A Nationwide Longitudinal Study. Diabetes Care.

[4] Wei J.N., Sung F.C., Lin C.C., Lin R.S., Chiang C.C., Chuang L.M. (2003). National surveillance for type 2 diabetes mellitus in Taiwanese children. JAMA.

[5] Van Name M.A., Cheng P., Gal R.L., Kollman C., Lynch J., Nelson B., Tamborlane W.V. (2020). Children and adolescents with type 1 and type 2 diabetes mellitus in the Pediatric Diabetes Consortium Registries: Comparing clinical characteristics and glycaemic control. Diabet. Med..

[6] Mulvaney S.A., Mudasiru E., Schlundt D.G., Baughman C.L., Fleming M., VanderWoude A., Russell W.E., Elasy T.A., Rothman R. (2008). Self-management in type 2 diabetes: The adolescent perspective. Diabetes Educ..

[7] Hagger V., Hendrieckx C., Sturt J., Skinner T.C., Speight J. (2016). Diabetes Distress Among Adolescents with Type 1 Diabetes: A Systematic Review. Curr. Diabetes Rep..

[8] Xu H., Verre M.C. (2018). Type 2 Diabetes Mellitus in Children. Am. Fam. Physician.

[9] World Health Organization Adolescent Health in the South-East Asia Region. https://www.who.int/southeastasia/health-topics/adolescent-health.

[10] Arnett J.J. (2000). Emerging adulthood. A theory of development from the late teens through the twenties. Am. Psychol..

[11] Feldman R.S. (2016). Development across the Life Span-Pearson.

[12] Auslander W.F., Sterzing P.R., Zayas L.E., White N.H. (2010). Psychosocial resources and barriers to self-management in African American adolescents with type 2 diabetes: A qualitative analysis. Diabetes Educ..

[13] Brouwer A.M., Salamon K.S., Olson K.A., Fox M.M., Yelich-Koth S.L., Fleischman K.M., Hains A.A., Davies W.H., Kichler J.C. (2012). Adolescents and type 2 diabetes mellitus: A qualitative analysis of the experience of social support. Clin. Pediatr..

[14] Salamon K.S., Brouwer A.M., Fox M.M., Olson K.A., Yelich-Koth S.L., Fleischman K.M., Hains A.A., Davies W.H., Kichler J.C. (2012). Experiencing type 2 diabetes mellitus: Qualitative analysis of adolescents’ concept of illness, adjustment, and motivation to engage in self-care behaviors. Diabetes Educ..

[15] Wong J.J., Addala A., Abujaradeh H., Adams R.N., Barley R.C., Hanes S.J., Iturralde E., Lanning M.S., Naranjo D., Tanenbaum M.L. (2020). Depression in context: Important considerations for youth with type 1 vs type 2 diabetes. Pediatr. Diabetes.

[16] Iturralde E., Rausch J.R., Weissberg-Benchell J., Hood K.K. (2019). Diabetes-Related Emotional Distress over Time. Pediatrics.

[17] Fisher L., Polonsky W.H., Hessler D. (2019). Addressing diabetes distress in clinical care: A practical guide. Diabet. Med..

[18] Bandura A. (1986). Social Foundations of thought and Actions: A Social Cognitive Theory.

[19] Bandura A. (1995). Self-Efficacy in Changing Societies.

[20] Wallston K.A., Rothman R.L., Cherrington A. (2007). Psychometric properties of the Perceived Diabetes Self-Management Scale (PDSMS). J. Behav. Med..

[21] Davidson M., Penney E.D., Muller B., Grey M. (2004). Stressors and self-care challenges faced by adolescents living with type 1 diabetes. Appl. Nurs. Res..

[22] Noser A.E., Huffhines L., Clements M.A., Patton S.R. (2017). Diabetes conflict outstrips the positive impact of self-efficacy on youth adherence and glycemic control in type 1 diabetes. Pediatr. Diabetes.

[23] Law G.U., Walsh J., Queralt V., Nouwen A. (2013). Adolescent and parent diabetes distress in type 1 diabetes: The role of self-efficacy, perceived consequences, family responsibility and adolescent-parent discrepancies. J. Psychosom. Res..

[24] Nouwen A., Urquhart L.G., Hussain S., McGovern S., Napier H. (2009). Comparison of the role of self-efficacy and illness representations in relation to dietary self-care and diabetes distress in adolescents with type 1 diabetes. Psychol. Health.

[25] Chen C.Y., Lo F.S., Wang R.H. (2020). Roles of Emotional Autonomy, Problem-Solving Ability and Parent-Adolescent Relationships on Self-Management of Adolescents with Type 1 Diabetes in Taiwan. J. Pediatr. Nurs..

[26] Faul F., Erdfelder E., Buchner A., Lang A.G. (2009). Statistical power analyses using G*Power 3.1: Tests for correlation and regression analyses. Behav. Res. Methods.

[27] Polonsky W.H., Anderson B.J., Lohrer P.A., Welch G., Jacobson A.M., Aponte J.E., Schwartz C.E. (1995). Assessment of diabetes-related distress. Diabetes Care.

[28] Polonsky W.H., Fisher L., Earles J., Dudl R.J., Lees J., Mullan J., Jackson R.A. (2005). Assessing psychosocial distress in diabetes: Development of the diabetes distress scale. Diabetes Care.

[29] Welch G.W., Jacobson A.M., Polonsky W.H. (1997). The Problem Areas in Diabetes Scale. An evaluation of its clinical utility. Diabetes Care.

[30] Huang M.F., Courtney M., Edwards H., McDowell J. (2010). Validation of the Chinese version of the Problem Areas in Diabetes (PAID-C) scale. Diabetes Care.

[31] Lin S.M., Lin S.L., Wu Y.C., Chang C.M., Wu H.L. (2011). Validity and reliability of a Chinese translation of a Perceived Diabetes Self-management Scale. J. Nurs. Healthc. Res..

[32] Ministry of Health and Welfare Body Mass Index for Children and Adolescents. https://www.hpa.gov.tw/Pages/Detail.aspx?nodeid=542&pid=9547.

[33] Kleinbaum D.G., Kupper L.L., Muller K.E., Nizam A. (1998). Applied Regression Analysis and Other Multivariable Methods.

[34] Copeland K.C., Zeitler P., Geffner M., Guandalini C., Higgins J., Hirst K., Kaufman F.R., Linder B., Marcovina S., McGuigan P. (2011). Characteristics of adolescents and youth with recent-onset type 2 diabetes: The TODAY cohort at baseline. J. Clin. Endocrinol. Metab..

[35] Goran M.I., Gower B.A. (2001). Longitudinal study on pubertal insulin resistance. Diabetes.

[36] Marshall W.A., Tanner J.M. (1970). Variations in Pattern of Pubertal Changes in Boys. Arch. Dis. Child..

[37] Marshall W.A., Tanner J.M. (1969). Variations in Pattern of Pubertal Changes in Girls. Arch. Dis. Child..

[38] Forsander G., Bogelund M., Haas J., Samuelsson U. (2017). Adolescent life with diabetes-Gender matters for level of distress. Experiences from the national TODS study. Pediatr. Diabetes.

[39] Islam M.R., Karim M.R., Habib S.H., Yesmin K. (2013). Diabetes distress among type 2 diabetic patients. Int. J. Med. Biomed. Res..

[40] Huynh E., Rand D., McNeill C., Brown S., Senechal M., Wicklow B., Dart A., Sellers E., Dean H., Blydt-Hansen T. (2015). Beating Diabetes Together: A Mixed-Methods Analysis of a Feasibility Study of Intensive Lifestyle Intervention for Youth with Type 2 Diabetes. Can. J. Diabetes.

[41] Chang L.Y., Li H.Y., Wei J.N., Chuang L.M. (2006). Type 2 diabetes and obesity in children and adolescents: Experience from studies in Taiwanese population. Curr. Diabetes Rev..

